# Marburg Virus VP30 Is Required for Transcription Initiation at the Glycoprotein Gene

**DOI:** 10.1128/mbio.02243-22

**Published:** 2022-08-23

**Authors:** Megan R. Edwards, Olivia A. Vogel, Hiroyuki Mori, Robert A. Davey, Christopher F. Basler

**Affiliations:** a Center for Microbial Pathogenesis, Institute for Biomedical Sciences, Georgia State University, Atlanta, Georgia, USA; b Department of Microbiology, Icahn School of Medicine at Mount Sinai, New York, New York, USA; c Department of Microbiology, National Emerging Infectious Diseases Laboratories, Boston University, Boston, Massachusetts, USA; Columbia University Medical College

**Keywords:** Marburg virus, RNA replication, filovirus, transcription

## Abstract

Marburg virus (MARV) is an enveloped, negative-sense RNA virus from the filovirus family that causes outbreaks of severe, frequently fatal illness in humans. Of the seven MARV proteins, the VP30 protein stands out because it is essential for viral growth but lacks a definitive function. Here, we used model MARV genome RNAs for one or two reporter genes and the MARV VP40, glycoprotein (GP), and VP24 genes to demonstrate that VP30 is dispensable for the transcription of some genes but critical for transcription reinitiation at the GP gene. This results in the loss of the expression of GP and downstream genes and the impaired production of infectious particles when VP30 is absent. Bicistronic minigenome assays demonstrate that the VP40 gene end/GP gene start junction specifically confers VP30 dependence. A region at the GP gene start site predicted to form a stem-loop contributes to VP30 dependence because the replacement of the GP stem-loop with corresponding sequences from the MARV VP35 gene relieves VP30 dependence. Finally, a Cys_3_-His zinc binding motif characteristic of filovirus VP30 proteins was demonstrated to be critical for reinitiation at GP. These findings address a long-standing gap in our understanding of MARV biology by defining a critical role for VP30 in MARV transcription.

## INTRODUCTION

Marburg virus (MARV) is a zoonotic pathogen that causes outbreaks of severe disease in humans. It belongs to the filovirus family, which also includes Ebola virus (EBOV), but is classified as belonging to a separate genus. The MARV and EBOV negative-sense RNA genomes contain seven genes, each of which encodes a distinct mRNA or, in the case of the EBOV glycoprotein (GP) gene, multiple mRNAs due to cotranscriptional editing by the viral polymerase (1).

Of the MARV genes and proteins, VP30 is notable because while its expression was required for the recovery of a recombinant MARV, and knockdown of VP30 with small interfering RNA (siRNA) reduced viral RNA and protein production, a specific VP30 function has yet to be defined (2, 3). In contrast to MARV VP30, EBOV VP30 has been demonstrated to regulate viral transcription. Best defined is the role of EBOV VP30 in the transcription of the nucleoprotein (NP) gene. This was first demonstrated by minigenome (MG) assays in which a model EBOV RNA for a reporter gene is coexpressed with the viral RNA polymerase complex, which consists of the NP, VP35, VP30, and large (L) proteins. In this assay, reporter gene expression is EBOV VP30 dependent (4). VP30 dependence is conferred by a stem-loop that can form at the NP gene transcription start site adjacent to the reporter gene (5). Disruption of the stem-loop renders EBOV minigenome transcription VP30 independent. The protranscription function of EBOV VP30 is regulated by its phosphorylation and requires that VP30 have an intact Cys_3_-His zinc binding motif and RNA binding activity (6–10). Other activities attributed to EBOV VP30 include facilitating reinitiation at more downstream genes and regulating the RNA editing of GP mRNAs (11, 12). Outside the filovirus family, the pneumovirus M2-1 proteins, which are transcription elongation factors and possess Cys_3_-His zinc binding motifs, are the only VP30 homologs identified (13).

When MARV transcription and replication were assessed using a minigenome assay, VP30 was dispensable, although the omission of VP30 modestly diminished reporter gene expression (4). Modulation of MARV VP30 phosphorylation also affects minigenome activity, and MARV VP30 can partially replace EBOV VP30 in an EBOV minigenome assay (14, 15). These data suggest that while MARV VP30 is not essential for minigenome assays that employ single reporter genes, it may still play a role in MARV RNA synthesis.

Filovirus transcription proceeds by a stop-start mechanism where transcription begins toward the 3′ end of the negative-sense viral genomic RNA at the NP gene (16). Transcription then proceeds from “left” to “right,” with the transcription of one gene terminating at a gene end signal, followed by reinitiation at the next gene start signal (16). Single-gene minigenome assays do not assess stops and starts across multiple genes and therefore do not address the full complexity of viral transcription. In the present study, based on a reporter gene system described previously for EBOV (17), we employed polycistronic model MARV genomic RNAs possessing one or two reporter genes and the MARV VP40, GP, and VP24 genes. By coexpressing the model genome with the viral polymerase complex, the system recapitulates the major steps in the MARV replication cycle, including the transcription of multiple viral genes. By employing these and bicistronic minigenome assays, we demonstrate a critical role of MARV VP30 in transcription reinitiation at the GP gene. By assigning a function to VP30, these data address a long-standing gap in the understanding of MARV replication and provide assays that can be used to further characterize MARV VP30 function.

## RESULTS

### Establishment of a transfection-based assay to model the MARV life cycle.

**FIG 1:**
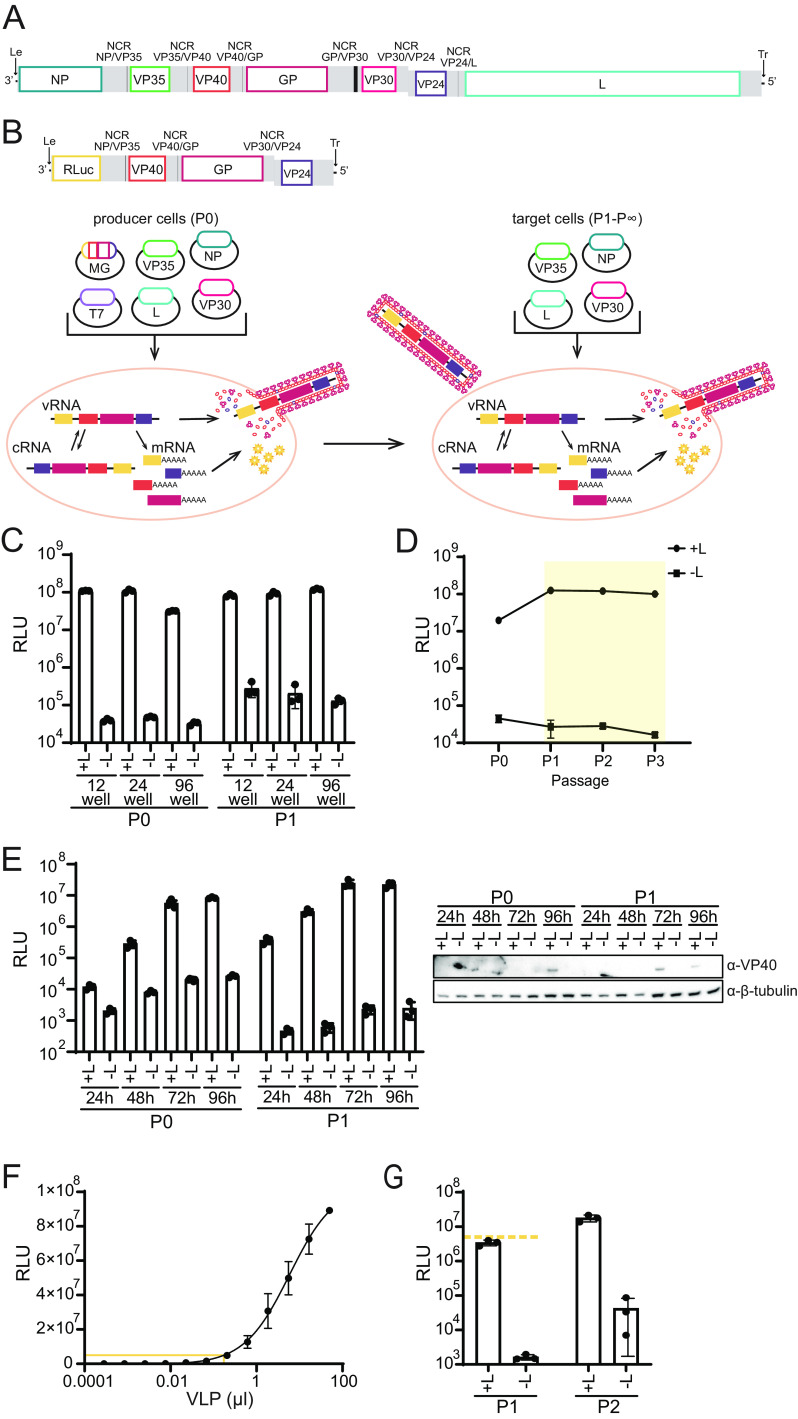
Development and characterization of a MARV tetracistronic minigenome and a transcription/replication-competent VLP assay. (A) Marburg virus genome organization. Le, leader; Tr, trailer. Gray indicates untranslated regions (UTRs), and black indicates intergenic regions. A gene overlap present in the VP30/VP24 NCR is indicated. (B, top) Schematic of the MARV tetracistronic minigenome (tMG) reporter. The leader and trailer of MARV flank open reading frames (ORFs) for *Renilla* luciferase (RLuc), VP40, GP, and VP24. The construct also includes the noncoding regions (NCRs) corresponding to the junctions of NP/VP35, VP40/GP, and VP30/VP24 in the MARV genome, as indicated. Gray indicates UTRs, and black indicates intergenic regions. (Bottom) To perform the transcription- and replication-competent virus-like particle (trVLP) assay, producer cells (P0) are transfected with the minigenome (MG) reporter and expression plasmids for T7 RNA polymerase (T7) and the viral polymerase complex components VP35, NP, VP30, and L. T7 RNA polymerase drives the initial transcription of the negative-sense RNA genome (viral RNA [vRNA]) from the MG plasmid, while the viral polymerase complex replicates the genome through a cRNA intermediate and transcribes mRNAs for *Renilla* luciferase, VP40, GP, and VP24. As all viral proteins are expressed from the tMG or protein expression plasmids, trVLPs that package the MG-nucleocapsid are produced. In P0, *Renilla* luciferase activity measures genome replication and transcription by the viral polymerase complex. trVLPs can infect target cells (P1 to P∞) that have been transfected with VP35, NP, VP30, and L. The resulting luciferase activity reflects a combination of polymerase activity in P0, assembly and budding of trVLPs from P0, and entry of trVLPs into target cells. Multiple infectious cycles can be modeled through repeated passaging of supernatants onto target cells where VP35, NP, VP30, and L expression plasmids have been provided. (C) Huh7 P0 cells were transfected in 12-. 24- and 96-well formats with plasmids to produce the tMG, T7 RNA polymerase, VP35, NP, and VP30, with L being either included (+L) or omitted (−L), as indicated. At 72 h posttransfection (hpt), *Renilla* luciferase activity was determined, and the supernatants were passaged onto P1 cells transfected with VP35, NP, VP30, and L. Luciferase activity was measured at 72 h postinfection (hpi). Error bars represent the standard deviations for triplicate samples. (D) Huh7 P0 cells were transfected in a 24-well format as described above for panel C in 24-well format, and at 72 hpt, luciferase activity was measured. The P0 supernatants were passaged onto P1 target cells, and luciferase activity was measured at 72 hpi. This was repeated for P2 and P3 target cells. The yellow-shaded area indicates the passage of trVLPs onto target cells transfected with all components of the polymerase complex. Error bars represent the standard deviations for triplicate samples. (E) Huh7 P0 cells were transfected as described above for panel C in a 96-well format, and luciferase activity was assessed at 24, 48, 72, and 96 hpt. The supernatants from 72 hpt were passaged onto P1 target cells, and luciferase activity was measured every 24 hpi from 24 to 96 hpi. Error bars represent the standard deviations for triplicate samples. Western blot analysis of cell lysates was performed for VP40 and β-tubulin expression. (F) Huh7 P1 target cells were infected with an increasing volume of trVLPs, and luciferase activity was measured at 72 hpi. The yellow line indicates the volume of trVLPs (0.18 μL) required to induce 5 × 10^6^ RLU. Error bars represent the standard deviations for duplicate experiments. (G) Huh7 P1 target cells were infected with 0.18 μL of trVLPs from panel F, in the presence or absence of L. Luciferase activity was measured at 72 hpi, and the supernatants were passaged onto P2 target cells that were assessed at 72 hpi. Error bars represent the standard deviations for triplicate experiments. The yellow dashed line indicates 5 × 10^6^ RLU.

To enable the study of essential viral functions, including the role of VP30, we sought to establish a MARV life cycle modeling assay, based on a previously described EBOV system (17). A construct was built to model the natural MARV genome (Fig. 1A). This model genome lacks the NP, VP35, VP30, and L genes that encode proteins required for MARV RNA synthesis and encodes a *Renilla* luciferase (RLuc) reporter, VP40, GP, and VP24 (Fig. 1B). The genes in the tetracistronic minigenome (tMG) are separated by non-protein-coding regions (NCRs) corresponding to the NP/VP35, VP40/GP, and VP30/VP24 gene junctions and include sequences that regulate the transcription of these viral genes. The ends of the genome correspond to the leader and trailer sequences necessary for the replication of the genome by the MARV polymerase complex. A T7 promoter abuts the trailer sequences, while a ribozyme and a T7 terminator are adjacent to the leader sequences. When introduced into producer (passage 0 [P0]) cells along with T7 RNA polymerase, the model genome is transcribed into a negative-sense RNA such that the 3′ and 5′ ends correspond to the ends of the authentic MARV genome. The coexpression of the viral VP35, VP30, NP, and L proteins leads to the replication and transcription of the model genome. The resulting *Renilla* luciferase expression from these P0 cells provides a measure of viral transcription and replication efficiency. Because all of the MARV proteins are expressed either from the tMG or from protein expression plasmids, virus-like particles (VLPs) that package the tMG RNA can be produced. These particles can be passaged onto new cells (passage 1 [P1]) that, if previously transfected to express the viral polymerase complex, should allow another round of replication and particle production. This transfection-based assay can be safely performed at biosafety level 2 and recapitulates MARV replication, gene expression, assembly, and infectivity (Fig. 1B).

The MARV transcription- and replication-competent VLP (trVLP) system works efficiently in Huh7 cells across 12-, 24-, and 96-well formats, as indicated by the robust luciferase signal in all of the samples that included a full complement of plasmids, including the MARV L protein (+L), at both P0 and P1 (Fig. 1C). To control for background luciferase levels, L was omitted (−L) and replaced with an empty vector for the initial transfection. Cells at subsequent steps of the assay were always transfected with the full complement of polymerase complex components. The luciferase signal remained stable across at least three passages (Fig. 1D). A time course of P0 and P1 activities showed an approximately 10-fold increase in luciferase activity in the presence of L every 24 h, between 24 and 72 h (Fig. 1E). Although the absolute signals can vary from experiment to experiment, a consistent signal (+L)-to-background (−L) ratio of between ~100 and 1,000 is obtained. Using a Western blot assay, VP40 expression was detected in P0 lysates at 96 h posttransfection (hpt) and in P1 lysates at 72 and 96 h postinfection (hpi). VP24 was undetectable in this assay. However, in a larger-plate format, it was detectable in the whole-cell lysate (WCL) and purified VLPs (see Fig. S1A in the supplemental material). Although several antibodies were tested, GP was not detectable by Western blotting in either format.

10.1128/mbio.02243-22.1 FIG S1 Further characterization of a MARV tetracistronic minigenome and the transcription/replication-competent VLP assay. (A) Huh7 P0 cells in a 6-well format were transfected with the tMG, T7, VP35, NP, VP30, and L. Luciferase activity was assessed at 72 hpt, and the supernatants were purified over a 20% sucrose gradient. The whole-cell lysate (WCL) and purified VLPs were analyzed by Western blotting for NP, VP40, and VP24 expression. (B) Huh7 P0 cells in a 24-well format were transfected with the tMG, T7, VP35, NP, VP30, and L, as indicated. At 72 hpt, luciferase activity was assessed, and the supernatants were passaged onto Huh7 or HEK293T P1 target cells transfected with VP35, NP, VP30, L, and Tim1, as indicated. P1 luciferase activity was determined at 72 hpi. In panels A and B, error bars represent the standard deviations for triplicate samples. Western blot assays of representative lysates from the trVLP transfections were performed using anti-Flag antibody to detect Flag-tagged Tim1 and anti-β-tubulin. Download FIG S1, PDF file, 0.7 MB. Copyright © 2022 Edwards et al. 2022 Edwards et al. https://creativecommons.org/licenses/by/4.0/ This content is distributed under the terms of the Creative Commons Attribution 4.0 International license.

A previous study using the equivalent EBOV trVLP system found that exogenous Tim1 expression was required for the most efficient trVLP infection of HEK293 cells, which do not express Tim1 (17). While Huh7 cells, used throughout this study, express Tim1, it was of interest to determine if exogenous Tim1 could further enhance the infection efficiency and whether it is required for MARV trVLP infection of HEK293T cells (18). To assess this, trVLPs were generated in Huh7 P0 cells, and the supernatants were passaged onto Huh7 or HEK293T P1 target cells pretransfected with all components of the polymerase complex and Tim1, as indicated (Fig. S1B). The exogenous expression of Tim1, which was confirmed by Western blotting, did not enhance the infection efficiency for either Huh7 or HEK293T cells (Fig. S1B). Initial transcription of the tMG in P0 cells is driven by the T7 polymerase, which has the potential to confound measurements of trVLP RNA levels. To use the assay in a T7-independent manner, a trVLP stock was generated from a P0 transfection, and the titer was determined on P1 target cells (Fig. 1F). Using this titration, the volume of VLPs required to reach the desired luciferase signal in P1 can be calculated, providing an additional level of control for the assay, akin to a specific multiplicity of infection with virus (Fig. 1F and G).

### VP30 is required for downstream transcription reinitiation.

**FIG 2:**
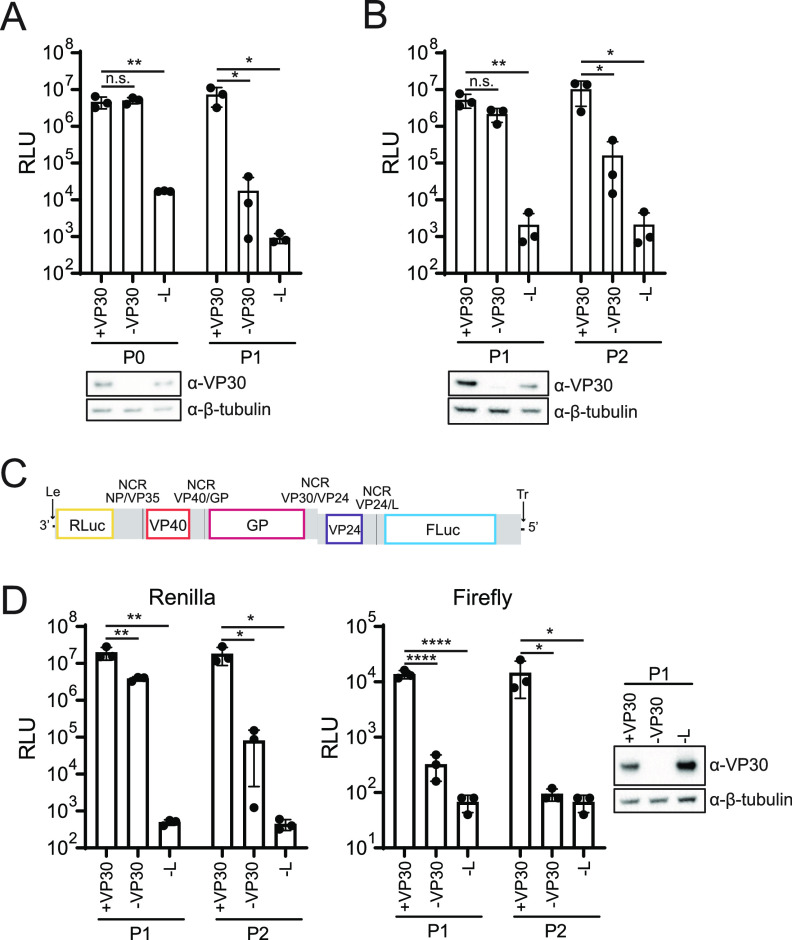
VP30 expression is required for downstream gene expression. (A) Huh7 P0 target cells in a 96-well format were transfected with the tMG, T7, VP35, NP, and, as indicated, VP30 and L. Luciferase activity was assessed at 72 hpt, and the supernatants were used to infect P1 target cells transfected with VP35, NP, VP30, and L. P1 luciferase activity was determined at 48 hpi. (B) trVLPs were used to infect P1 target cells in a 96-well format transfected with VP35, NP, and, as indicated, VP30 and L. Luciferase activity was determined at 72 hpi, and the supernatants were passaged onto P2 target cells transfected with VP35, NP, VP30, and L. P2 luciferase activity was assessed at 48 hpi. (C) Schematic of the pentacistronic MG (pMG). Le, leader; Tr, trailer. Gray indicates untranslated regions (UTRs), and black indicates intergenic regions. Firefly luciferase (FLuc), preceded by the NCR between VP24 and L, was added to the tMG as a fifth gene. (D) Pentacistronic trVLPs (ptrVLPs) were used to infect P1 target cells in a 96-well format transfected as described above for panel B. *Renilla* and firefly luciferase activities were determined at 72 hpi. The supernatants were used to infect P2 target cells transfected as described above for panel B, and luciferase activity was assessed at 48 hpi. In panels A, B, and D, Western blot assays were performed for VP30 and β-tubulin. Error bars represent the standard deviations for triplicate experiments, and statistical significance was determined for the indicated comparisons by one-way analysis of variance (ANOVA), followed by Dunnett’s test (****, *P* < 0.0001; **, *P* < 0.01; *, *P* < 0.05; n.s., no significance).

To determine how VP30 impacts the MARV trVLP assay, P0 cells were transfected with the tMG and the components of the polymerase complex, omitting VP30 or L, as indicated. The supernatants were subsequently passaged onto P1 target cells (Fig. 2A). As previously seen with the monocistronic minigenome (mMG), VP30 expression was not required for P0 *Renilla* luciferase activity. However, VP30 was required for efficient P1 luciferase activity. Western blot analysis confirmed VP30 expression where expected. The assay was repeated using a trVLP stock to infect P1 target cells, leaving out VP30 or L, as indicated (Fig. 2B). The absence of VP30 in the infected P1 target cells resulted in a small, nonsignificant decrease in *Renilla* luciferase, while a more substantial ~100-fold decrease in luciferase activity was detected in P2 cells, comparing “−VP30” to “+VP30.” This recapitulates the results of the P0-to-P1 assay.

To determine if VP30 affects the expression of downstream open reading frames (ORFs), a pentacistronic minigenome (pMG) reporter was constructed, inserting firefly luciferase as the final ORF in the reporter (Fig. 2C). Using the pMG for P0 transfection, pentacistronic trVLPs (ptrVLPs) were generated and used to infect P1 target cells that were pretransfected with all polymerase complex components, omitting VP30 or L, as indicated (Fig. 2D). As expected for the final gene in the pMG, the expression level of firefly luciferase was consistently lower than that of *Renilla* luciferase. The P1 supernatants were then transferred to P2 target cells transfected with the complete set of polymerase complex components. While *Renilla* luciferase activity was slightly decreased in P1 in the absence of VP30, a 100-fold reduction occurred in the P2 target cells. In contrast, in the absence of VP30, firefly luciferase levels decreased in P1 nearly to those of the “−L” control, and minimal expression was detected in P2 cells. Together, these data suggest that VP30 plays a role in infectious particle production and the expression of genes downstream of *Renilla* luciferase.

**FIG 3:**
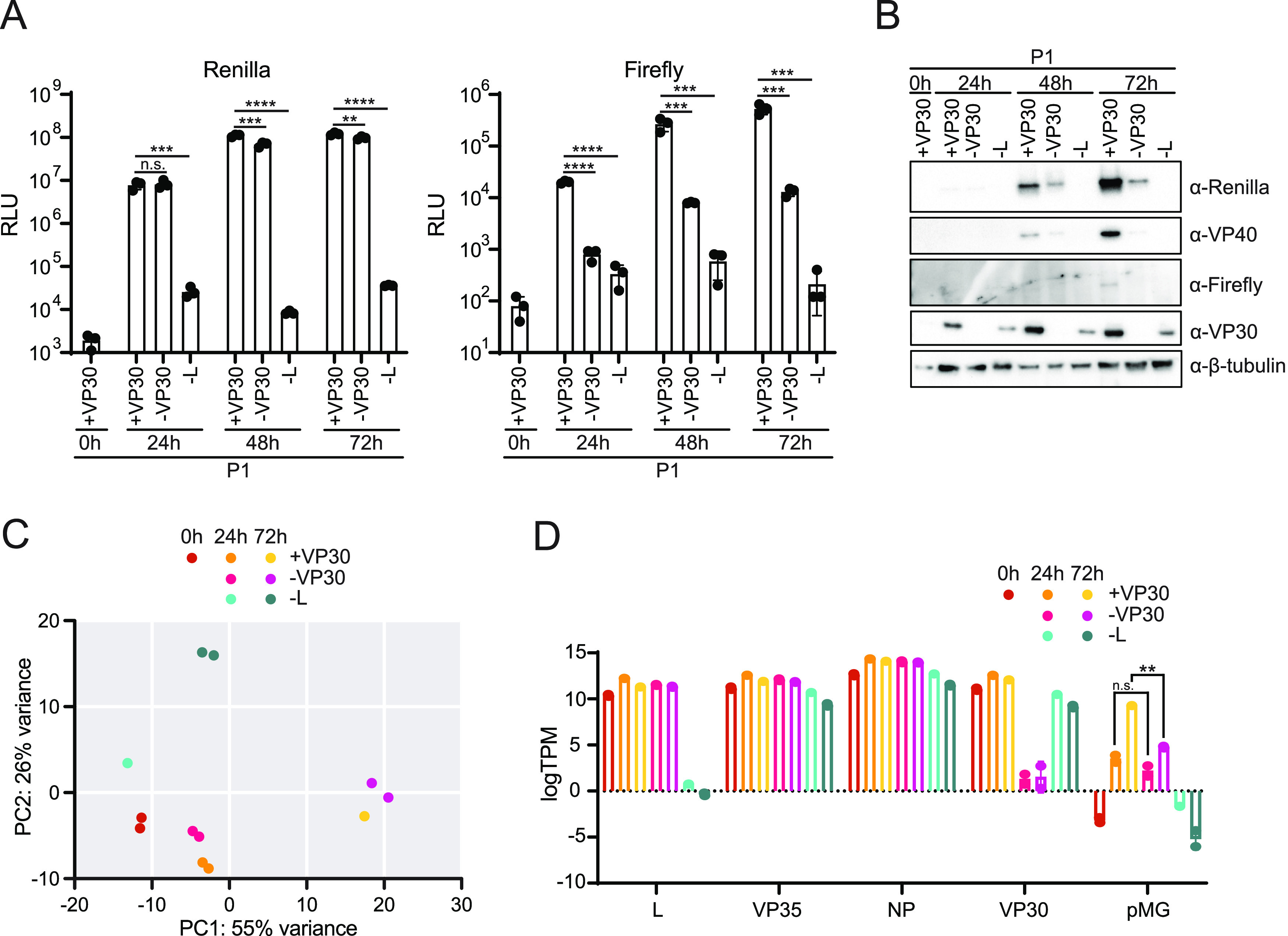
RNAseq analysis of the role of VP30 in the MARV life cycle. (A) Huh7 P1 target cells in a 24-well format transfected with VP35, NP, and, as indicated, VP30 and L were infected with ptrVLPs. Luciferase activity was assessed at 0, 24, 48, and 72 hpi, and error bars represent the standard deviations for triplicate experiments. Statistical significance was determined for the comparisons indicated at each time point by one-way ANOVA, followed by Dunnett’s test (****, *P* < 0.0001; ***, *P* < 0.001; **, *P* < 0.01; n.s., no significance). (B) Western blot analysis of *Renilla* luciferase, VP40, firefly luciferase, VP30, and β-tubulin expression at each time point. (C) Principal-component analysis (PCA) of RNAseq samples performed by using DESeq2. (D) Log transcripts per million (logTPM) aligned to L, VP35, NP, VP30, and pMG sequences. Error bars represent the standard deviations for duplicate samples. Statistical significance for the indicated comparisons in the pMG was determined by one-way ANOVA, followed by the Šidák correction (**, *P* < 0.01; n.s., no significance).

To determine how VP30 exerts its positive effect on gene expression, RNA sequencing (RNAseq) was performed on P1 target cells. The P1 cells were pretransfected with NP, VP35, VP30, and L, omitting VP30 or L, as indicated. *Renilla* and firefly luciferase activities were measured at 0, 24, 48, and 72 hpt (Fig. 3A). Over the time course, minimal differences in *Renilla* luciferase activity were detected whether VP30 was present or absent, although these differences reached statistical significance at 48 and 72 hpi. Firefly luciferase activity, however, was significantly decreased in the absence of VP30, with a >10-fold reduction at all time points. Western blotting of the lysates confirmed the expression of VP30 where expected, although the expression levels were higher in the presence of L than in its absence (Fig. 3B). *Renilla* luciferase and VP40 were also detected, with protein levels being higher in +VP30 than in −VP30 samples, and not expressed in −L samples, as expected. Firefly luciferase was detected by Western blotting at 72 hpi in the presence of VP30 only.

RNA was extracted from the corresponding samples for +VP30 at 0, 24, and 72 hpi and −VP30 and −L at 24 and 72 hpi. RNAseq was performed on duplicate samples, except for single replicates for −L at 24 hpi and +VP30 at 72 hpi, due to RNA quality issues in their duplicate samples. Principal-component analysis (PCA) demonstrated distinct profiles in the presence and absence of L and across the three time points (Fig. 3C). Interestingly, samples in which VP30 was included or omitted clustered near each other at 24 and 72 hpi, suggesting that few differences in host gene expression result from VP30 expression. Alignment of the RNAseq reads to the transfected polymerase complex components confirmed the expected absence of L and VP30 expression in their respective samples (Fig. 3D). Mapping the reads onto the pMG sequence demonstrated an increase in minigenome transcripts over the time course in the presence of VP30. At 24 hpi, pMG transcript levels were comparable in the absence and presence of VP30, but by 72 hpi, the levels were substantially higher in the presence of VP30. pMG expression in samples where L was not expressed resembled that at the 0-hpi time point, confirming the lack of transcriptional activity in the absence of the viral polymerase (Fig. 3D).

**FIG 4:**
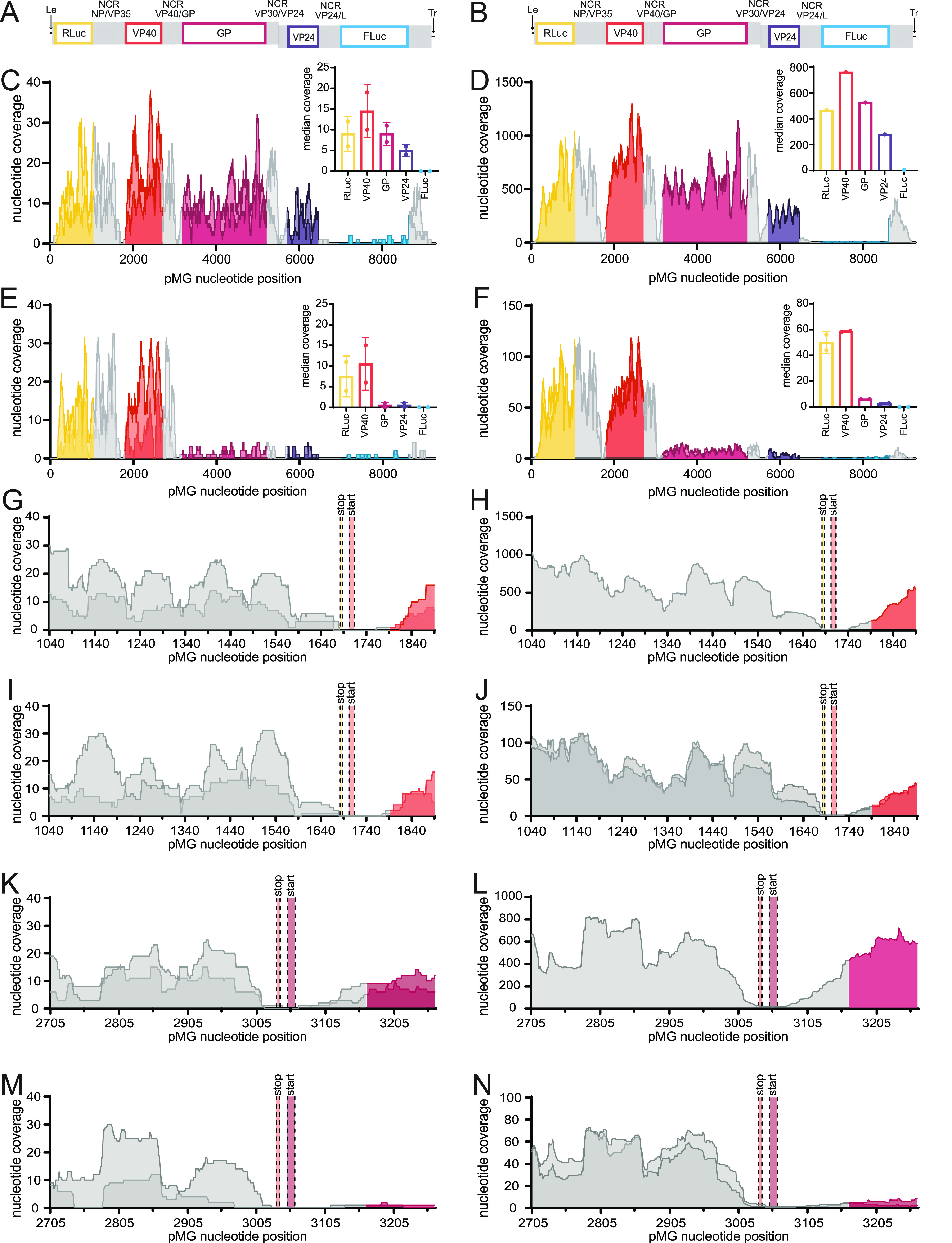
VP30 is required for transcription downstream of the GP gene start site. (A and B) pMG organization. Le, leader; Tr, trailer. Gray indicates untranslated regions (UTRs), and black indicates intergenic regions. A gene overlap present in the VP30/VP24 NCR is indicated. (C to F) Nucleotide coverage at each pMG position for +VP30 at 24 hpi (C), +VP30 at 72 hpi (D), −VP30 at 24 hpi (E), and −VP30 at 72 hpi (F). Duplicate samples are shown in panels C, E, and F by different shading. A single replicate is shown in panel D. The inset graphs represent the median nucleotide coverage for each of the five ORFs, and error bars represent the standard deviations for duplicate samples. (G to J) Nucleotide coverage across the NP/VP35 NCR and the 5′ end of VP40 (pMG nt positions 1040 to 1890) at +VP30 at 24 hpi (G), +VP30 at 72 hpi (H), −VP30 at 24 hpi (I), and −VP30 at 72 hpi (J). (K to N) Nucleotide coverage across the VP40/GP NCR and the 5′ end of GP (pMG nt positions 2705 to 3264) at +VP30 at 24 hpi (K), +VP30 at 72 hpi (L), −VP30 at 24 hpi (M), and −VP30 at 72 hpi (N). Duplicate samples are shown in panels G, I to K, M, and N. A single replicate is shown in panels H and L.

At 24 hpi, similar nucleotide coverages across the RLuc and VP40 genes were demonstrated in the presence and absence of VP30 (Fig. 4A, C, and E). However, in the absence of VP30, there were 18- and 10-fold decreases in the median coverages of GP and VP24, respectively, compared with the samples that contained VP30 (Fig. 4C and E). At 72 hpi, the read coverage in the presence of VP30 increased by approximately 50-fold over 24 hpi (Fig. 4C and D). In the absence of VP30 at 72 hpi, the median nucleotide coverages of *Renilla* luciferase and VP40 were 9- and 13-fold lower, respectively (Fig. 4D and F). Strikingly, the omission of VP30 again resulted in drastic reductions in the median nucleotide coverages of GP and VP24, with 88- and 111-fold decreases, respectively (Fig. 4D and F). An additional notable finding for all of the samples was a decrease in reads aligning to the VP24/L NCR and the firefly luciferase mRNA, suggesting either a deletion of this region of the genome during replication or contamination by the tMG plasmid (Fig. 4A to D). Fortunately, this does not impact the interpretation of the results of the experiment as the effect of removing VP30 from the system is readily apparent at the VP40/GP junction.

A close examination of read coverage at the NP gene stop and VP35 gene start sites that lie between the *Renilla* luciferase and VP40 genes in the pMG demonstrates that transcription reinitiation at the VP35 start site was not influenced by the presence or absence of VP30 at 24 hpi (Fig. 4G and I). At 72 hpi, the ~10-fold decrease in nucleotide coverage in the absence of VP30 is apparent; however, reinitiation was not further affected (Fig. 4H and J). In contrast, in the absence of VP30 at either 24 or 72 hpi, little transcription reinitiation was detected at the GP gene start site (Fig. 4K to N). Therefore, VP30 is required for the efficient transcription reinitiation and expression of genes downstream of the GP gene start site. The overall decrease in nucleotide coverage in the absence of VP30 at 72 hpi further suggests a role for VP30 that is independent of GP transcription reinitiation as infection progresses.

**FIG 5:**
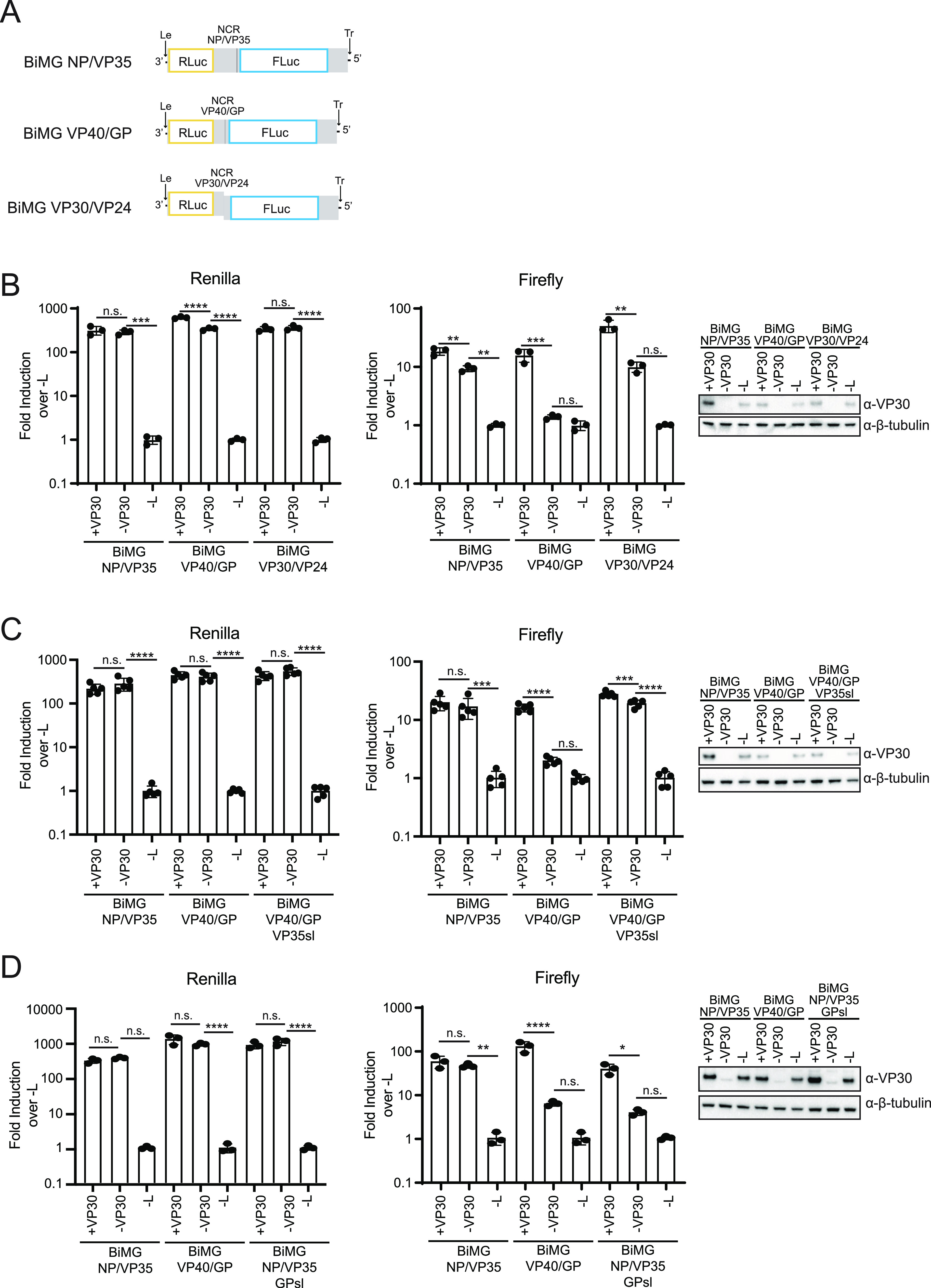
VP30 is required for transcription reinitiation at the GP gene start site. (A) Schematic of bicistronic minigenome (BiMG) constructs in which *Renilla* luciferase (RLuc) and firefly luciferase (FLuc) ORFs were separated by the 3′ and 5′ noncoding regions from the MARV NP and VP35 (NCR NP/VP35), MARV VP40 and GP (NCR VP40/GP), or MARV VP30 and VP24 (NCR VP30/VP24) genes. Le, leader; Tr, trailer. Gray, untranslated regions (UTRs); black, intergenic regions. (B) The indicated BiMGs were transfected into Huh7 cells in a 96-well format with T7, VP35, NP, and, as indicated, VP30 and L (included or omitted). Luciferase activity was assessed at 72 hpt. Error bars represent the standard deviations for triplicate samples, and statistical analysis was performed in a BiMG reporter for the indicated comparisons by one-way ANOVA, followed by the Šidák correction (****, *P* < 0.0001; ***, *P* < 0.001; **, *P* < 0.01; n.s., no significance). Levels of VP30 and β-tubulin were assessed by Western blotting. (C) Huh7 cells were transfected as described above for panel B with the indicated BiMG reporters. BiMG VP40/GP VP35sl has the predicted stem-loop from the GP 5′ NCR replaced with the predicted stem-loop from the VP35 5′ NCR. Luciferase activity was assessed as described above, and error bars represent the standard deviations for five samples. Statistical analysis and Western blot assays were performed as described above. (D) Huh7 cells were transfected as described above for panel B with the indicated BiMG reporters. BiMG NP/VP35 GPsl has the predicted stem-loop from the VP35 5′ NCR replaced with the predicted stem-loop from the GP 5′ NCR. Luciferase activity was assessed at 48 hpt, and error bars represent the standard deviations for three samples. Statistical analysis and Western blot assays were performed as described above.

To confirm the dependence and/or independence of viral gene start sites on VP30 expression, five bicistronic MG (BiMG) constructs were generated. These constructs each contain the viral leader and trailer regions flanking the ORFs for *Renilla* luciferase and firefly luciferase. They differ in that the reporter genes were separated by the NP/VP35 NCR, the VP40/GP NCR, the VP30/VP24 NCR, the VP35/VP40 NCR, or the GP/VP30 NCR (Fig. 5A and Fig. S2A). Therefore, for each of the constructs, *Renilla* luciferase activity utilizes the NP gene start site, while firefly luciferase activity is initiated from the VP35, GP, VP24, VP40, or VP30 gene start site. Following the transfection of the BiMG reporters, T7 RNA polymerase, and the viral polymerase complex components into Huh7 cells, *Renilla* and firefly luciferase activities were measured (Fig. 5B and Fig. S2B). As expected, minimal differences in *Renilla* luciferase activity were detected in the presence and absence of VP30 expression across the five reporters. Firefly luciferase activity following the NP/VP35 NCR was slightly reduced in the absence of VP30. Nonetheless, this construct maintained significantly higher expression levels than the −L control, indicating that transcription reinitiation at the VP35 start site does not require VP30 expression, a finding that corresponds to the nucleotide coverage determined by RNAseq (Fig. 4I and J). In contrast, the lack of VP30 resulted in a nearly complete loss of firefly luciferase activity for the VP40/GP construct, in agreement with the decrease in nucleotide coverage following the GP gene start site in the RNAseq experiment (Fig. 4M and N and Fig. 5B). A more intermediate phenotype was demonstrated by firefly luciferase expression following the VP24 gene start site, with a significant decrease in luciferase activity in the absence of VP30, which remained ~10-fold above that of the −L control (Fig. 5B). In the absence of VP30, the firefly luciferase signal following the VP40 start site demonstrated a modest but statistically significant reduction, whereas that following the VP30 start site was not significantly affected (Fig. S2B). These data suggest that different gene start sites exhibit different degrees of dependence on VP30, with the GP start site being the most sensitive to the absence of VP30.

10.1128/mbio.02243-22.2 FIG S2 Different degrees of VP30 dependence at the VP40 and VP30 gene start sites. (A) Schematic of bicistronic minigenome (BiMG) constructs in which *Renilla* and firefly luciferase ORFs were separated by the 3′ and 5′ noncoding regions from the MARV VP35 and VP40 (NCR VP35/VP40) or the MARV GP and VP30 (NCR GP/VP30) genes. Le, leader; Tr, trailer. Gray indicates untranslated regions (UTRs), and black indicates intergenic regions. (B) The indicated BiMGs were transfected into Huh7 cells in a 96-well format with T7, VP35, NP, and, as indicated, VP30 and L (included or omitted). Luciferase activity was assessed at 48 hpt. Error bars represent the standard deviations for triplicate samples, and statistical analysis was performed for the indicated comparisons (in a given BiMG reporter) by one-way ANOVA, followed by the Šidák correction (****, *P* < 0.0001; ***, *P* < 0.001; **, *P* < 0.01; n.s., no significance). Levels of VP30 and β-tubulin were assessed by Western blotting. Download FIG S2, PDF file, 0.5 MB. Copyright © 2022 Edwards et al. 2022 Edwards et al. https://creativecommons.org/licenses/by/4.0/ This content is distributed under the terms of the Creative Commons Attribution 4.0 International license.

Previous work predicted stem-loop structures to be present within each of the MARV gene start sites (16, 19). To determine whether VP30 dependence is conferred by the putative GP stem-loop, a BiMG was constructed where the GP stem-loop sequence was swapped for that of VP35 (BiMG VP40/GP VP35sl) (Fig. 5C). Again, *Renilla* luciferase activity was not affected by the presence or absence of VP30 expression. As previously seen, the expression of firefly luciferase following the VP35 gene start site was independent of VP30 expression, while that following the GP gene start site was VP30 dependent. Strikingly, the replacement of the GP stem-loop sequence with that of VP35 resulted in firefly luciferase expression independent of VP30 expression. A reciprocal experiment was then performed, where the putative stem-loop at the GP start site was inserted in the place of the VP35 stem-loop in the NP/VP35 construct (BiMG NP/VP35 GPsl) (Fig. 5D). In this experiment, as before, *Renilla* luciferase was unaffected by the absence of VP30 for all constructs tested. However, firefly luciferase expression from the NP/VP35 GPsl construct was diminished compared to that of the NP/VP35 control and closely resembled that of the VP40/GP construct. These data point to the predicted GP stem-loop as conferring VP30 dependence.

**FIG 6:**
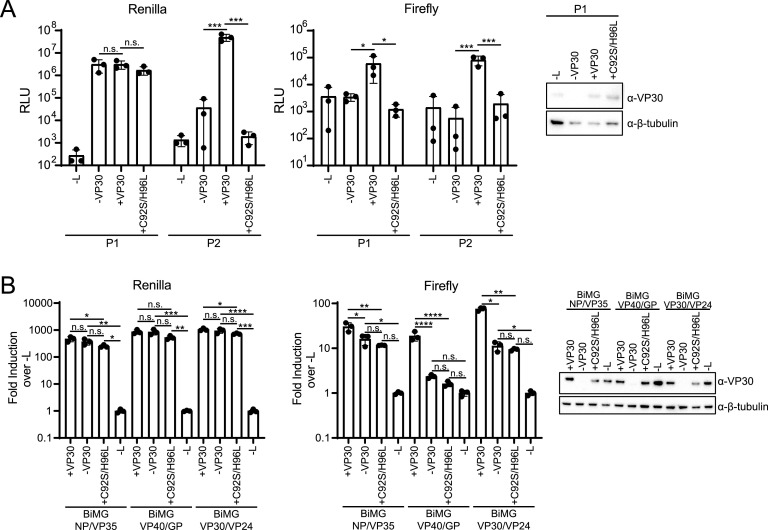
The VP30 zinc binding motif is required for transcription reinitiation activity. (A) Huh7 P1 target cells in a 96-well format were transfected with VP35, NP, and, as indicated, VP30, VP30 C92S/H96L, and L. The transfected cells were infected with ptrVLPs. Luciferase activity was determined at 72 hpi, and the supernatants were passaged onto P2 target cells transfected with VP35, NP, VP30, and L. P2 luciferase activity was assessed at 48 hpi. Error bars represent the standard deviations for triplicate samples, and statistical significance was determined by one-way ANOVA, followed by the Šidák correction (***, *P* < 0.001; *, *P* < 0.05; n.s., no significance). Levels of VP30, VP30 C92S/H96L, and β-tubulin were determined by Western blotting. (B) The indicated BiMGs were transfected into Huh7 cells in a 96-well format with T7, VP35, NP, and, as indicated, VP30, VP30 C92S/H96L, and L. Luciferase activity was determined at 72 hpi, and error bars represent the standard deviations for triplicate samples. Statistical significance for the indicated comparisons in a BiMG reporter was determined by one-way ANOVA, followed by the Šidák correction (****, *P* < 0.0001; ***, *P* < 0.001; **, *P* < 0.01; *, *P* < 0.05; n.s., no significance). Levels of VP30, VP30 C92S/H96L, and β-tubulin were determined by Western blotting.

A zinc binding motif is shared among filovirus VP30 proteins, and for EBOV VP30, it is required for activity from an EBOV mMG reporter (10). To clarify the role of this motif in MARV VP30, a zinc binding motif mutant, VP30 C92S/H96L, was generated, and a P1-to-P2 ptrVLP assay was performed (Fig. 6A). *Renilla* luciferase activities in P1 target cells were similar regardless of whether VP30, VP30 C92S/H96L, or no VP30 was expressed (Fig. 6A). However, only supernatants from wild-type VP30-transfected cells resulted in *Renilla* luciferase activity in P2 target cells. In addition, firefly luciferase activity was detected in P1 target cells only when wild-type VP30 was expressed (Fig. 6A). This demonstrates that the VP30 zinc binding motif is necessary for the efficient expression of downstream MARV genes and the production of ptrVLPs.

To determine if the VP30 zinc binding motif is required for transcription reinitiation, BiMG assays were performed using the BiMG NP/VP35, BiMG VP40/GP, and BiMG VP30/VP24 reporter constructs. Across the three BiMGs, modest effects on *Renilla* luciferase activity were detected in the absence of VP30 or when the VP30 C92S/H96L mutant was expressed (Fig. 6B). Although firefly luciferase activity decreased in the absence of VP30 or when the VP30 C92S/H96L mutant was expressed with either BiMG NP/VP35 or BiMG VP30/VP24, these constructs maintained ~10-fold activity over the −L control (Fig. 6B). In contrast, firefly luciferase expression was decreased to nearly background control levels for BiMG VP40/GP when VP30 was not expressed or the zinc binding mutant was used, indicating that the zinc binding motif of VP30 is required for its transcription reinitiation activity at the GP gene start site.

## DISCUSSION

The data described above demonstrate that VP30 is required for the transcription of the MARV GP gene and that its absence also generally reduces viral mRNA levels over time, likely explaining why VP30 is required for MARV growth (2, 4). In contrast, transcription is VP30 independent at the NP gene start signal and the VP35 gene start signal. Because the attenuation of GP transcription also impairs downstream transcription, the impact of VP30 on initiation at the VP24 and firefly luciferase genes in the pMG cannot be readily addressed. However, the bicistronic MGs allow the testing of individual gene start sites, and in this context, VP24 and VP40 transcription initiation is modestly impacted when VP30 is absent, while transcription initiation at VP30 is not affected. Whether initiation at the L gene start depends on VP30 remains to be determined.

Given that the requirement for VP30 at the EBOV NP transcription start site depends on RNA secondary structure, it was of interest to determine whether a similar mechanism might be at play in the MARV system. Each of the MARV transcription start sites has the potential to form secondary structures (20). The replacement of the predicted stem-loop at the MARV GP transcription start site with the corresponding sequences from the MARV VP35 transcription start site eliminated the need for VP30, whereas the reciprocal experiments rendered the VP35 start site VP30 dependent. This demonstrates that specific sequences confer VP30 dependence and suggests that RNA secondary structure may play a role, as is the case for EBOV VP30 (5, 21).

MARV may have evolved such that GP transcription has a specific requirement for VP30. What purpose this regulation serves is unclear. In EBOV, NP transcription depends on VP30 (5). As the first gene transcribed, impaired transcription of NP impacts the transcription of all downstream genes. In this way, the functional status of EBOV VP30 can be envisioned to serve as a switch between transcription and replication (6, 22, 23). In MARV, it is possible that GP expression requires regulation. For EBOV, cotranscriptional editing of GP gene mRNAs determines the ratio of GP to secreted GP (24–26). It has been proposed that this strategy prevents cytotoxicity due to excessive GP production in EBOV-infected cells (24). It is also notable that EBOV VP30 regulates GP editing (12). MARV VP30 dependence could provide a mechanism to prevent excessive MARV GP expression.

Whether and how the MARV VP30 transcription function is regulated should also be defined. For EBOV, dephosphorylated VP30 promotes transcription, whereas phosphorylated VP30 impairs transcription but promotes genome replication and interaction with NP (6, 7). Phosphorylation also alters EBOV VP30 RNA binding, suggesting mechanisms by which phosphorylation regulates activity (22). The EBOV VP30 phosphorylation state is determined by its interaction with cellular serine-arginine protein kinase 1 (SRPK1) and SRPK2, its interaction with NP, and the recruitment by NP of the PP2A-B56 protein phosphatase (27, 28). Select host proteins with PPXPXY motifs, including RBBP6 and hnRNP L, compete with NP for binding to EBOV VP30, impair VP30 phosphorylation, and decrease viral gene expression (29, 30). MARV VP30 is also a phosphoprotein, and MARV VP30 phosphorylation modestly impairs the transcription of a monocistronic MARV minigenome (15). The dependence of both EBOV and MARV VP30s on an intact Cys_3_-His zinc binding motif provides another functional similarity (10). It will be of interest to determine whether MARV VP30 function is regulated by means similar to those affecting EBOV VP30. The demonstration of a definitive function and the development of assays to monitor this function provide opportunities to address these questions.

## MATERIALS AND METHODS

### Cells.

HEK293T cells (ATCC CRL-3216) and Huh7 cells (a generous gift from the Gordan laboratory at the University of California at San Francisco) were maintained in Dulbecco’s modified Eagle medium (DMEM) supplemented with 10% fetal bovine serum (FBS) and cultured at 37°C with 5% CO_2_.

### Plasmids.

pCAGGS MARV NP, VP35, VP30, L, and T7 RNA polymerase (T7) plasmids have been previously described (31). The mutant VP30 C92S/H96L was generated by overlapping PCR and cloned into pCAGGS. pCMV3 Flag-tagged Tim1 was purchased from Sino Biological (catalog number HG11051-NF). The tetracistronic minigenome (tMG) construct was synthesized by GenScript and inserted into the pM1 vector. From 5′ to 3′, it contains a T7 terminator, a ribozyme, the viral leader, the NP 5′ untranslated region (UTR), the *Renilla* luciferase coding sequence, the NP/VP35 NCR, the VP40 coding sequence, the VP40/GP NCR, the GP coding sequence, the VP30/VP24 NCR, the VP24 coding sequence, the L 3′ UTR, the trailer sequence, and a T7 promoter. The MARV sequences correspond to those under GenBank accession number DQ217792.1. To generate the pentacistronic minigenome (pMG), the tMG was digested with BglII (found at nucleotides [nt] 346 to 341 in the VP24 coding sequence) and NotI (flanks the T7 promoter at the 3′ end). The VP24/L NCR was synthesized by GenScript, and the firefly coding sequence was obtained by PCR from the pGL4.18[luc2P/Neo] vector (Promega). Three fragments were generated by PCR, which spanned (i) the end of the VP24 coding sequence, (ii) the VP24/L NCR, and (iii) the firefly coding sequence, L 3′ UTR, trailer, and T7 promoter. Using NEBuilder HiFi DNA assembly (New England BioLabs [NEB]), these fragments and the digested tMG were assembled. Sequences were confirmed by Sanger sequencing. Bicistronic minigenome (BiMG) constructs were synthesized using the pMG as the template and overlapping PCR to generate a fragment containing the T7 terminator, the leader, the NP 5′ UTR, *Renilla* luciferase, and either the NP/VP35, VP40/GP, VP30/VP24, VP35/VP40, or GP/VP30 NCR. A second fragment containing firefly luciferase, the L 3′ UTR, the trailer, and the T7 promoter was generated by PCR from the pMG template. The two fragments were assembled with the pM1 vector using NEBuilder HiFi DNA assembly (NEB). BiMG VP40/GP VP35sl was synthesized by GenScript and contained the putative VP35 stem-loop sequence (GAAGAATATTAAAGATTTTCTTTAATATTCAGA) in place of the GP stem-loop sequence (GAAGAACATTAATTGCTGGGTAAAAGTGATTAATTTCTTT).

### Generation of ptrVLP stocks.

P0 Huh7 cells (1 × 10^7^ cells/15-cm dish) were transfected with L at 10 μg, VP35 at 0.625 μg, VP30 at 0.625 μg, NP at 5 μg, pMG at 2.5 μg, and T7 at 2.5 μg. At 72 hpt, the supernatant was harvested and clarified by centrifugation at 3,000 rpm for 5 min at 4°C. ptrVLPs were precipitated with a polyethylene glycol (PEG) virus precipitation kit (catalog number K904; BioVision), aliquoted, flash-frozen, and stored at −80°C. To determine the luciferase activity of the ptrVLP stock, P1 Huh7 target cells (3 × 10^4^ cells/well) in a 96-well format were transfected with L at 166 ng, VP35 at 10.4 ng, VP30 at 10.4 ng, and NP at 83.3 ng per well using TransIT-LT1 (Mirus Bio) (3:1 reagent-to-DNA ratio). At 24 hpt, cells were infected in duplicate with a 2-fold dilution series (50 μL to 0.02 μL). Medium was changed to fresh DMEM with 10% FBS at 24 hpi, and luciferase activity was assessed at 72 hpi using a dual-luciferase assay (Promega) and an EnVision plate reader.

### Purification of trVLPs.

P0 Huh7 cells in a 6-well format were transfected with L at 1,000 ng, VP35 at 62.5 ng, VP30 at 62.5 ng, NP at 500 ng, tMG at 250 ng, and T7 at 250 ng in triplicate. At 72 hpt, the supernatants were harvested and clarified by centrifugation at 1,000 rpm at 4°C for 10 min. The clarified supernatant was overlaid onto a 20% sucrose cushion in NTE buffer (10 mM NaCl, 10 mM Tris [pH 7.5], 1 mM EDTA [pH 8.0]) and ultracentrifuged in a Beckman SW-41 rotor at 36,000 rpm for 2 h at 4°C. After centrifugation, the supernatant was aspirated, and trVLPs were resuspended in NP-40 lysis buffer (50 mM Tris [pH 7.5], 280 mM NaCl, 0.5% Igepal CA-630, 0.2 mM EDTA, 2 mM EGTA, 10% glycerol). The luciferase activity of the cells was assessed by a dual-luciferase assay (Promega).

### MARV trVLP and ptrVLP assays. (i) P0-to-P1 assays.

P0 Huh7 cells (96-well format, 3 × 10^4^ cells/well; 24-well format, 1.5 × 10^5^ cells/well; 12-well format, 3 × 10^5^ cells/well) were transfected using TransIT-LT1 (Mirus Bio) (3:1 reagent-to-DNA ratio) with L at 166 ng, VP35 at 10.4 ng, VP30 at 10.4 ng, NP at 83.3 ng, tMG/pMG at 41 ng, and T7 at 41 ng per well in the 96-well format; L at 500 ng, VP35 at 31.25 ng, VP30 at 31.25 ng, NP at 250 ng, tMG/pMG at 125 ng, and T7 at 125 ng per well in the 24-well format; or L at 1,000 ng, VP35 at 62.5 ng, VP30 at 62.5 ng, NP at 500 ng, tMG/pMG at 250 ng, and T7 at 250 ng in the 12-well format, omitting L and VP30 as indicated. Forty-eight hours after the transfection of P0 cells, P1 Huh7 cells were transfected with L, VP35, VP30, and NP at concentrations equivalent to those at P0. Where included, 125 ng of the Flag-Tim1 plasmid was transfected. Twenty-four hours after P1 transfection (72 h after transfection of P0), medium was removed from P1 transfected cells, and the P0 supernatants (96-well format, 50 μL; 24-well format, 125 μL; 12-well format, 250 μL) were transferred. Unless otherwise indicated, P0 luciferase activity was measured at 72 hpt using a dual-luciferase assay (Promega) and read on an EnVision plate reader. At 24 hpi, medium from P1 was changed to fresh DMEM with 10% FBS, and the infection was allowed to proceed for an additional 24 h, at which time P1 luciferase activity was measured. The assays were performed in triplicate; error bars represent the standard deviations for the triplicate.

### (ii) P1-to-P2 assays.

P1 target Huh7 cells in a 96-well format were transfected with L, VP35, VP30, and NP at the above-mentioned concentrations, omitting L or VP30, as indicated. At 24 hpt, cells were infected with a volume of trVLPs required to induce *Renilla* luciferase activity at 10^6^ to 10^7^ relative luciferase units (RLU) at 72 hpi. After 6 h of infection, the inoculum was removed, and fresh DMEM with 10% FBS was added. Forty-eight hours after the infection of P1 cells, P2 Huh7 cells were transfected with L, VP35, VP30, and NP at the above-described concentrations. Twenty-four hours after the transfection of P2 target cells, medium was removed, and 50 μL of the supernatant from P1 was transferred. P1 luciferase activity was measured at 72 hpi. Twenty-four hours after the infection of P2 target cells, medium was changed to fresh DMEM with 10% FBS, and after an additional 24 h, P2 luciferase activity was measured. The assays were performed in triplicate; error bars represent the standard deviations for the triplicate.

### Bicistronic minigenome assay.

Huh7 cells (3 × 10^5^ cells/well) in a 96-well format were transfected with L at 166 ng, VP35 at 10.4 ng, VP30 at 10.4 ng, NP at 83.3 ng, T7 at 41 ng, and the indicated BiMG at 41 ng per well. At 72 or 48 hpt, luciferase activity was measured using a dual-luciferase assay (Promega) and read on an EnVision plate reader. The assays were performed in either three or five replicates, as indicated; error bars represent the standard deviations for the samples.

### RNAseq experiment.

Huh7 cells (1.5 × 10^5^ cells/well) in a 24-well format were transfected using TransIT-LT1 (Mirus Bio) (3:1 reagent-to-DNA ratio) with VP35 at 31.25 ng, NP at 250 ng, VP30 at 31.25 ng, and L at 500 ng per well, omitting VP30 and L, as indicated, in five replicates. At 24 hpt, all samples were infected. At 24 hpi, medium was changed to fresh DMEM with 10% FBS. At the indicated time points after infection, luciferase activity was assessed in three replicates with the lysate stored at −20°C for Western blot analysis The remaining two replicates were harvested in TRIzol and stored at −80°C until RNA isolation using the Direct-zol RNA miniprep kit (Zymo Research). RNA quality and concentrations were determined using a 2100 bioanalyzer (Agilent Genomics). Libraries were prepared using the NEB poly(A) mRNA magnetic isolation kit and the NEB Ultra II RNA directional kit. Multiplexed libraries were subjected to single-end 100-bp sequencing using the NovaSeq 6000 platform (Illumina).

Data analysis was performed using the Galaxy instance of Georgia State University (GSU). RNA reads were trimmed using TrimGalore with an average Phred score cutoff of 30 and a minimum length of 50 bp. FastQC was used to generate quality reports. Reads that passed quality control (1.73 × 10^7^ to 2.93 × 10^7^ reads/sample) were aligned to a custom reference genome (Homo_sapiens.GRCh38.dna.primary_assembly.fa) and a custom annotation file (Homo_sapiens.GRCh38.103.gtf) containing VP35, VP30, NP, L, and pMG sequences using HiSat2, with percent alignments of between 95.2 and 97.3% per sample (32). Gene-level read counts were generated using featureCounts, counting reads overlapping exons, and differentially expressed genes (DEGs) were assessed using edgeR (33, 34). The principal-component diagram was generated using DESeq2. Read coverage for the pMG was extracted from the alignment files using the SAMtools depth function and plotted in Prism 9 (GraphPad).

### Western blot assays.

Lysates were run on 10% Bis-Tris polyacrylamide gels (Invitrogen) and transferred to polyvinylidene difluoride (PVDF) membranes (Bio-Rad). Membranes were probed with the indicated antibodies, developed using Western Lightning Plus ECL (Perkin-Elmer) or the SuperSignal West Femto substrate (Thermo Scientific), and imaged on a ChemiDoc MP imaging system (Bio-Rad).

### Antibodies.

Rabbit anti-β-actin (catalog number 3700) was purchased from Cell Signaling Technology. Rabbit anti-β-tubulin (catalog number T8328), mouse antiluciferase (catalog number L2164), and rabbit anti-Flag (catalog number F7425) were purchased from Sigma-Aldrich. Anti-*Renilla* luciferase (catalog number ab185926) was purchased from Abcam. Rabbit anti-MARV NP (catalog number 0303-012) and mouse anti-MARV VP40 (clone 6B1) (catalog number 0203-016) were purchased from IBT BioServices. Rabbit anti-MARV VP24 (peptide 230-REHSQMEKGQPLNLTQ-259) and anti-MARV VP30 (peptides 4-PRGRSRTRNHQVTPTIYHETQLPSK-28 and 258-CESSISVQASYDHFILPQSQGK-279) antibodies to the peptides indicated in parentheses were generated by Pacific Immunology.

### Statistical analysis.

Statistical analysis was performed using GraphPad Prism 9, with significance being determined as indicated in the figure legends. Data points were considered significantly different if the *P* value was <0.05.

### Data availability.

The GenBank BioProject accession number for the RNA sequencing data reported in this paper is PRJNA750472 (35).
