# Supplementary figures and images for: Marburg Virus VP30 Is Required for Transcription Initiation at the Glycoprotein Gene
Source: mBio. 2022 Aug 23;13(5):e02243-22. doi: 10.1128/mbio.02243-22 (PMC9601197; doi:10.1128/mbio.02243-22)

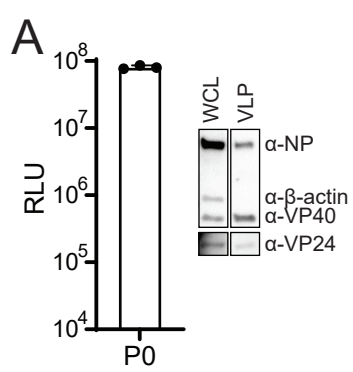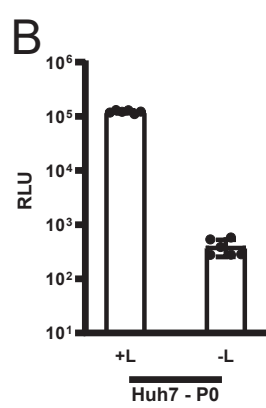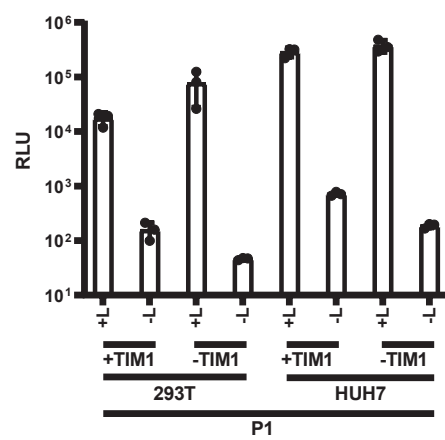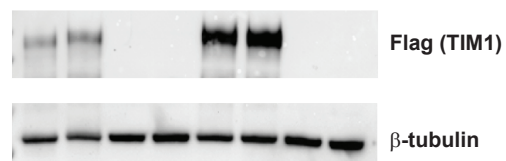

Supplement: FIG S1 [file mbio.02243-22-s0001.pdf]

# A

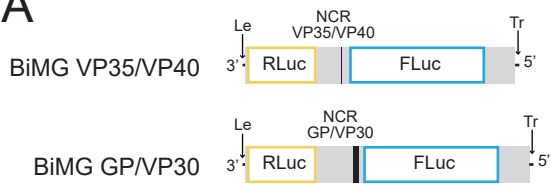

# B

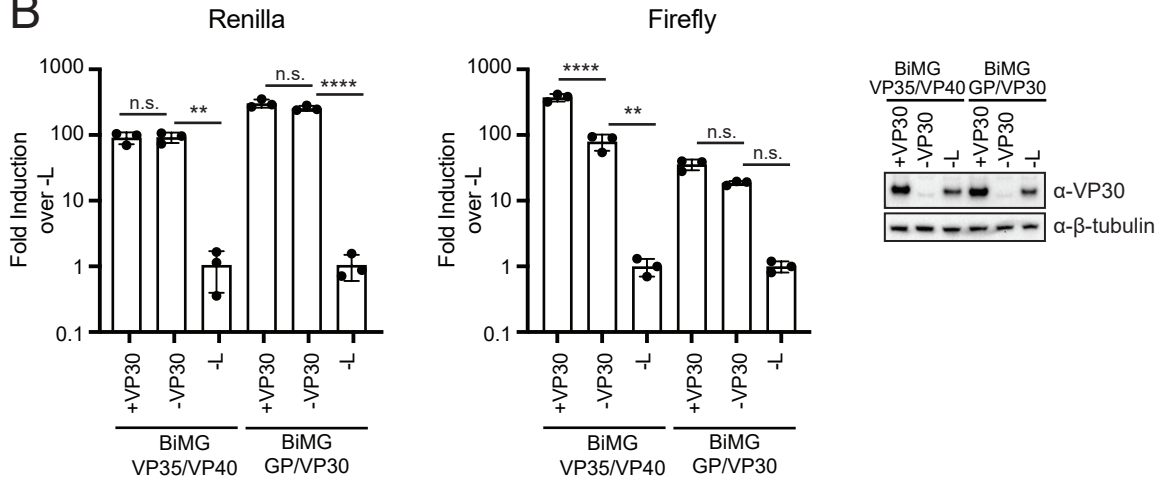

Supplement: FIG S2 [file mbio.02243-22-s0002.pdf]
